# Understanding Older Adults Motivations and Preferences for a Medication Adherence App

**DOI:** 10.1093/geroni/igab046.3539

**Published:** 2021-12-17

**Authors:** Olivia Kupiec, Maurita Harris, Wendy Rogers

**Affiliations:** 1 University of Illinois Urbana Champaign, Champaign, Illinois, United States; 2 University of Illinois at Urbana-Champaign, Champaign, Illinois, United States; 3 University of Illinois Urbana-Champaign, Champaign, Illinois, United States

## Abstract

As the percentage of older adults with hypertension continues to increase, medication adherence remains low. However, medication adherence can potentially be improved through the use of medication reminder apps. Medication reminder apps contain numerous features that enable older adults to remember to take their medication, such as providing alerts to take their medication, reminding them when to refill their prescription, and more. Despite being aware of these apps, many older adults lack the motivation needed to use them continuously. We recruited 9 participants (60 years or older) who currently take medication for a chronic condition. Using a mixed-methods approach, we gathered quantitative survey data using the TechSAge Demographic Background, Motivation, and Behavior Change Technique Questionnaires). Qualitative data were gathered through a semi-structured interview that asked questions about general motivations and preferences in addition to engaging participants in co-designing a medication adherence app. Results from the interview were analyzed through a thematic analysis that identified comprehension and preferences of older adults in medication reminder app usage. We tested five different intrinsic motivation factors, and results indicate older adults are most motivated intrinsically due to perceived choice, perceived competence, value/usefulness, effort/importance, and pressure/tension. We also tested five factors of extrinsic motivation, and results indicate older adults are most motivated extrinsically due to introjected regulation, reward-driven, external regulation, compliance, and identification. These data provide insights to guide the design of medication reminder apps to support older adults in the self-management of their chronic conditions.

